# Continuous in situ measurements of volcanic gases with a diode-laser-based spectrometer: CO_2 _and H_2_O concentration and soil degassing at Vulcano (Aeolian islands: Italy)

**DOI:** 10.1186/1467-4866-8-5

**Published:** 2007-04-20

**Authors:** Maurizio De Rosa, Gianluca Gagliardi, Alessandra Rocco, Renato Somma, Paolo De Natale, Giuseppe De Natale

**Affiliations:** 1 CNR, Istituto Nazionale di Ottica Applicata, Sezione di Napoli, Via Campi Flegrei 34, I-80078 Pozzuoli (NA), Italy; 2 INGV, Osservatorio Vesuviano, Via Diocleziano 328, I-80124 Napoli, Italy

## Abstract

We report on a continuous-measurement campaign carried out in Vulcano (Aeolian islands, Sicily), devoted to the simultaneous monitoring of CO_2 _and H_2_O concentrations. The measurements were performed with an absorption spectrometer based on a semiconductor laser source emitting around a 2-*μ*m wavelength. The emitted radiation was selectively absorbed by two molecular ro-vibrational transitions specific of the investigated species. Data for CO_2 _and H_2_O concentrations, and CO_2 _soil diffusive flux using an accumulation chamber configuration, were collected at several interesting sampling points on the island (Porto Levante beach- PLB, Fossa Grande Crater – FOG- and Valley of Palizzi, PAL). CO_2_/H_2_O values, measured on the ground, are very similar (around 0.019 (± 0.006)) and comparable to the previous discrete detected values of 0.213 (Fumarole F5-La Fossa crater rim) and 0.012 (Fumarole VFS – Baia Levante beach) obtaid during the 1977–1993 heating phase of the crater fumaroles.

In this work much more homogeneous values are found in different points of the three sites investigated. The field work, although carried out in a limited time window (25th–28th August 2004), pointed out the new apparatus is suitable for continuous gas monitoring of the two species and their ratios, which are important geochemical indicators of volcanic activity, for which other reliable continuous monitoring systems are not yet available.

## Background

Continuous monitoring of gases released in volcanic areas, emitted as fumaroles or diffusive degassing from soils, can give an insight for a deeper understanding of the volcanic dynamics and its impact on global climate and human activities [1,2]. The presence of numerous chemical species released in air and their variation vs. time can be related to chemical reactions of magma with host rocks and circulating fluids, occurring during its uprise. CO_2 _and H_2_O are molecular species which play a major role in volcanic dynamics. In fact, while water vapour can be related to an increasing heat flow caused by magma uprise movements, the volcanic CO_2 _is in general very abundant in magma but is less reactive than other gases during the interaction with acquifers and rocks [3]. The selection of these two species is based on their common use in volcanology and on their potential in providing useful information regarding volcano degassing dynamics. The CO_2 _is hence the best indicator of new deep magma supply, because its increase in concentration and flow at ground reflects increased exsolution from deep sources. In addition, since the CO_2 _is by far the most abundant gas component in volcanic fluids, the ratio between CO_2 _and H_2_O closely represent the gas/water ratio in the aquifers, which is sensitive to temperature increase in the deeper systems, which can result, for instance, by rising of hot fluids or magma [4].

Even though in the past years different techniques have been applied to monitoring of volcanic gases, e.g., mass spectrometry and gas cromatography, they are generally performed in laboratory, after collecting sample gases on field. No reliable techniques for long-term, continuous geochemical monitoring with near-laboratory precision do actually exist. Discrete on-site field measurements are generally restricted to CO_2 _soil diffusive flux [5]. Remote sensing techniques were developed in the last years for several gas species, such as Differential Optical Absorption Spectroscopy (DOAS) [6], Correlation Spectroscopy (COSPEC) [7], Light Detection And Ranging (LIDAR) [8], Fourier Transform Infrared (FTIR) Spectroscopy [9,10]. Eddy-covariance [11] (EC) micrometereological method can be alternative solutions, for CO_2 _flux, allowing measurements over large gas emission areas. Some of these methods are able to detect few trace species (e.g. SO_2_, HCl, HF) while H_2_S, CO_2 _and H_2_O, main constituents of volcanic gases, are spectroscopically hard to determine, therefore the corresponding volcanogenic emission rates can only be derived indirectly. More recently, continuous gas and temperature measurements have been performed using a set of analytical instruments to monitor volcanic gases over weeks [12-14].

Laser spectroscopy has been shown to be a powerful technique for field, non-invasive and real-time gas detection, based on the selective absorption of laser radiation by the molecules of the target gas [15,16]. Most molecules of interest have fundamental and overtone absorption bands in the infrared region of the electromagnetic spectrum. However, in the spectral region below 2 *μ*m the outstanding progress in the telecommunication technologies made available laser sources, like distributed feed-back (DFB) semiconductor lasers, which are ideal for the development of high-resolution, fast-response spectrometers. In previous works we applied this technology to monitor CO_2 _and H_2_O in volcanic fumaroles, using a dual-wavelength approach [17], delivering a beam with 1.57 and 1.39 *μ*m diode-laser radiation to a 20-cm-long cell placed upon the emitting fumarole by means of optical fibers. The dual-wavelength spectrometer enabled us to measure water vapor concentration with a 3% accuracy, whereas it was quite inaccurate for carbon dioxide measurements [18]. Subsequently, we implemented a new absorption spectrometer using a single room-temperature 2-*μ*m DFB diode laser for real-time measurement of both CO_2 _and H_2_O concentration [19,20]. At this wavelength, a considerably larger fractional absorption can be detected, due to the occurrence of stronger ro-vibrational transitions. The measurement technique was based on recording of direct absorption profiles of a gas sample contained in a multiple-reflection cell with a path-length of 50 m. Remote operation was still feasible using telecom-type optical fibers. Laboratory tests showed that it was possible to achieve a short-term reproducibility over several tens of minutes and an overall accuracy below 1% for measurements on ambient CO_2_. Simultaneous measurements of the CO_2 _and H_2_O concentrations mantain similar accuracy levels [20,21].

After the first field tests [20], the spectrometer has been upgraded and in this paper, we present the results of a measurement campaign carried out in August 2004 in Vulcano (Aeolian islands, Sicily). Vulcano, due to the long period of geochemical anomalies and seismicity [22,23], and the presence of high CO_2 _degassing [24-27], represents an ideal test site for the new spectroscopic apparatus, both for concentration and flux measurements. Further, the presence at some sites of fumaroles with very aggressive (hot and acid) components allow to test the apparatus and optics also in extreme conditions of operation. The volcanic CO_2 _discharges occur both from fumaroles and as diffuse soil emanations form the volcanic edifices [28].

### Geological setting of Vulcano fumaroles and soil degassing composition

The Vulcano Island is one of the most active volcanoes of the Mediterranean area; since its last explosive eruption, which occurred in 1888–1890 from the Fossa crater, the volcano has experienced fumarolic activity, mostly located along the northern rim of this crater [22]. Owing to a potentially pre-eruptive crisis begun in late 1977, numerous papers have been focused during the last 25 years on understanding the volcanic system at Volcano Island and on volcanic surveillance. Chemical composition changes of the fumarolic gases have been monitored for two crater fumaroles (FA and Fll) by Capasso et al. from the 1988 to 1991 [29]. The fumaroles studied showed an increase in temperature, ranging from 590°C to a maximum of 650°C for the FA fumarole. Moreover, the He/N_2_, He/CO_2 _and He/S ratios showed large variations with a general increase of helium, nitrogen, carbon dioxide and total sulphur contents and indicating a general trend like He>N_2 _>CO_2 _>total S. The behaviour of HCl concentration in the fumarole FA is opposite respect to those of He, N_2 _CO_2 _SO_2_. In average the variability of the gas composition at Vulcano have been explained [23] as an indicator of different degree of mixing processes between a Deeper Magmatic Component (DMC) and a Shallow Hydrotermal Component (SHC). Taking into account both non-reactive gases (e.g., N_2_, and He) and main constituents (i.e., H_2_O, and CO_2_), the maximum contribution of the DMC occurred in the summer of the 1988 while the maximum fraction of the SHC in fumarolic fluids was recorded through 1989. The following progressive increase in CO_2 _concentration through 1990 indicates an increasing DMC contribution, likely due to an energetic seismic event, which should have caused intense fracturation and permeability increase; it was followed by its decrease during the 1991, and finally followed, during the 1992, by an increase of the DMC contribution [23]. During the 1993–1995 period [30] monitoring of three types of sampled manifestations at Vulcano (Crater fumaroles, Baia di Levante and SIP, Palizzi and P4MAX) showed the occurrence of mixing between different end-members (crater hottest fumaroles, cooler shallower fluids, atmospheric component).

Gurrieri and Valenza have reported a diffusive CO_2 _gas monitoring at Vulcano island inserting in the soil a pipe opened at the base and measuring the dynamic concentration of the CO_2 _which is proportional to the ϕCO2
 MathType@MTEF@5@5@+=feaafiart1ev1aaatCvAUfKttLearuWrP9MDH5MBPbIqV92AaeXatLxBI9gBaebbnrfifHhDYfgasaacH8akY=wiFfYdH8Gipec8Eeeu0xXdbba9frFj0=OqFfea0dXdd9vqai=hGuQ8kuc9pgc9s8qqaq=dirpe0xb9q8qiLsFr0=vr0=vr0dc8meaabaqaciaacaGaaeqabaqabeGadaaakeaaiiGacqWFvpGAdaWgaaWcbaGaee4qamKaee4ta80aaSbaaWqaaiabikdaYaqabaaaleqaaaaa@3207@, unless a conversion factor, depending on the experimental device, working conditions, physical characteristics of the soil [31]. Since the end of 1984, several campaigns have been carried out using the latter method in the area of Vulcano Porto and in the area surrounding the cone of La Fossa [32,33], reporting values of flux as high as 8·10^-4 ^cm/s. Carapezza and Diliberto [34] have measured helium and CO_2 _soil degassing respectively using a mass spectrometer in the lab and using an IR spectrometer, directly in field, with an accuracy for CO_2 _concentration of 100 ppm. The average concentration of CO_2 _measured in the 1989–1991 in fifty-one different points shows a general increasing trend with fluctuations, from 1989 to the end of 1991, with a maximum value at the end of 1990. Moreover during this campaign it was evidenced that some points, located on the southern base of the cone (Grotta Palizzi) and on the W and NW cone slope (Forgia Vecchia) show higher values than those found in Vulcano Porto area. CO_2 _fluxes measured since the 1989 [33] suggest an increasing trend since the end of 1989 (~0.9·10^-4 ^cm/s per area unit) to the end of 1991 (~4.9·10^-4 ^cm/s per area unit), decreasing during the 1992–1993 period (~1.2·10^-4 ^cm/s per area unit). It is worth of note that the observed temporal variations are not influenced by seasonal variation of atmospheric parameters. This means that measurements of CO_2 _soil degassing can constitute an useful indicator of deep dynamics in case of further evolution of the activity in the island whereas the crater fumaroles get inaccessible. Moreover, the collected data at specific points (Point 4) during the 1990–1992 years interval seem to suggest that a feeding system was active and enriched in He and CO_2_, probably connected to La Fossa volcano [35], whereas elsewhere in the surveyed area He concentration was controlled by the extent of mixing between air and exhaling CO_2_. Continuous gas monitoring of CO_2 _in soils [36] carried out at different areas of the Vulcano island shows that the mean values of CO_2 _degassing in the most representative areas were lower than those of the previous year, in agreement with the observation carried out in the area of Vulcano Porto during the monthly surveys [33].

Accumulation chamber methodology used during a campaign in the period April-July 1996 in the upper part of the Fossa cone [25] measured a total output of 200 td^-1 ^of CO_2 _which corresponds to approximately 1000 Mgd^-1 ^of steam. In order to obtain the ϕCO2
 MathType@MTEF@5@5@+=feaafiart1ev1aaatCvAUfKttLearuWrP9MDH5MBPbIqV92AaeXatLxBI9gBaebbnrfifHhDYfgasaacH8akY=wiFfYdH8Gipec8Eeeu0xXdbba9frFj0=OqFfea0dXdd9vqai=hGuQ8kuc9pgc9s8qqaq=dirpe0xb9q8qiLsFr0=vr0=vr0dc8meaabaqaciaacaGaaeqabaqabeGadaaakeaaiiGacqWFvpGAdaWgaaWcbaGaee4qamKaee4ta80aaSbaaWqaaiabikdaYaqabaaaleqaaaaa@3207@ in the plains adjacent to the western and southern slopes of the Fossa volcanic edifice and in the isthmus separating Porto di Levante from Porto di Ponente, Chiodini et al. [26] measured emissions from 0.2 to 2900 gm^-2 ^d^-1 ^for a total amount of diffuse CO_2 _output of 75.3 Mgd^-1^. A survey on fifteen measurements of ϕCH4
 MathType@MTEF@5@5@+=feaafiart1ev1aaatCvAUfKttLearuWrP9MDH5MBPbIqV92AaeXatLxBI9gBaebbnrfifHhDYfgasaacH8akY=wiFfYdH8Gipec8Eeeu0xXdbba9frFj0=OqFfea0dXdd9vqai=hGuQ8kuc9pgc9s8qqaq=dirpe0xb9q8qiLsFr0=vr0=vr0dc8meaabaqaciaacaGaaeqabaqabeGadaaakeaaiiGacqWFvpGAdaWgaaWcbaGaee4qamKaeeisaG0aaSbaaWqaaiabisda0aqabaaaleqaaaaa@31FD@, ϕCO2
 MathType@MTEF@5@5@+=feaafiart1ev1aaatCvAUfKttLearuWrP9MDH5MBPbIqV92AaeXatLxBI9gBaebbnrfifHhDYfgasaacH8akY=wiFfYdH8Gipec8Eeeu0xXdbba9frFj0=OqFfea0dXdd9vqai=hGuQ8kuc9pgc9s8qqaq=dirpe0xb9q8qiLsFr0=vr0=vr0dc8meaabaqaciaacaGaaeqabaqabeGadaaakeaaiiGacqWFvpGAdaWgaaWcbaGaee4qamKaee4ta80aaSbaaWqaaiabikdaYaqabaaaleqaaaaa@3207@ and temperature was conducted at Porto di Levante [24] obtaining respectively: 0.007 to 3.9 g m^-2^d^-1^, 6.4 to 12000 gm^-2^d^-1^, and 20 to 100°C. Carapezza and Granierii [37] have proposed a comparison between active Dynamic Concentration and passive Accumulation Chamber methods to measure the carbon dioxide soil flux at the La Fossa areas of Vulcano Island. This work showed that the active methods overestimate the CO_2 _flux, and the results are proportional to it only in high-flux zones. Moreover, the carbon dioxide soil flux values were controlled in proximity of active gas-releasing fractures and in presence of changes in barometric pressure and soil permeability.

### Experimental setup

The Portable Diode Laser Spectrometer (PDLS) is based on a diode laser emitting infrared radiation around 2 *μ*m, where different ro-vibrational transition of CO_2 _and H_2_O occur. The PDLS has been previously described and characterized in details in [19-21]. Here we recall the principle of operation and the major features, mainly discussing the changes made in order to improve both the robustness of the system as well as its compactness, as required for a field instrument.

The spectrometer is made by three parts: the laser breadboard, the detection bench and the electronics. A 30 × 40 cm^2 ^ultra-light breadboard (10-kg-weight) hosts a DFB semiconductor laser (Sensor Unlimited) emitting at 1997 nm. The emitted radiation is collimated and coupled to a 30-m-long single-mode optical fiber by means of two stealing mirrors and a input lens which focus the ligth on one fiber end. In this way we are able to steadly couple about 10% of the incoming power.

With respect to the previous version of the spectrometer a factor 3 in the weight of the laser breadboard has been gained. The use of the fiber allows to keep the laser and the controlling electronics in a safe place, far from the sampling point, where environmental conditions can often be rather hostile and compromise a normal operation. The radiation is guided by the optical fiber to the detection bench, where it is collimated and injected by lenses and tilting mirrors into an open-path multipass Herriott cell. This cell is made by two gold-coated spherical mirrors, 50-mm-diameter and ~400-mm curvature radius, rigidly aligned and bolt 400 mm apart in the central part of the detection bench. All the lenses were coated with anti-reflective layer for reducing the occurrence of fringes which could deteriorate the absorption profile. A rectangular aperture (30 × 30 cm^2^) at the bottom of the bench allows the sampled gas flowing between the mirrors. The total optical path of the cell is 20 m long. The radiation transmitted by the cell is finally detected by a preamplified extended InGaAs detector. Also the detection bench has been modified, as a consequence of the experience of a previous field campaign (2002–2003) [20]: all the external surfaces are made of a titanium alloy, resulting in fact not only ligther compared to stainless steel, but also more resistant to acid compounds emitted in volcanic areas. As a result of experience for the previous campaigns, it was in fact observed that the mechanical components are the ones most suffering the hostile ambient, while the optics are not severely affected even after several days of operation. In order to vary the height of the breadboard with respect to the soil, the bench is equipped with extensible legs. The sides of the detection breadboard are protected to avoid external disturbances such as wind and dust. Then, a removable cover closes the upper part and allows also accumulation measurements in the detection chamber. In this way an evaluation of gas fluxes (ϕCO2
 MathType@MTEF@5@5@+=feaafiart1ev1aaatCvAUfKttLearuWrP9MDH5MBPbIqV92AaeXatLxBI9gBaebbnrfifHhDYfgasaacH8akY=wiFfYdH8Gipec8Eeeu0xXdbba9frFj0=OqFfea0dXdd9vqai=hGuQ8kuc9pgc9s8qqaq=dirpe0xb9q8qiLsFr0=vr0=vr0dc8meaabaqaciaacaGaaeqabaqabeGadaaakeaaiiGacqWFvpGAdaWgaaWcbaGaee4qamKaee4ta80aaSbaaWqaaiabikdaYaqabaaaleqaaaaa@3207@ soil) emitted by the soil can be performed.

The electronics and a laptop computer (LC) operate the laser, acquire the absorption signals and process them in real time. The laser temperature is actively stabilized providing a coarse tuning of its emission wavelength, then the frequency of the laser is continously scanned by sweeping its injection current with a triangular current ramp (2-Hz scan rate) around an offset constant value. The scanned frequency range is chosen in such a way to probe simultaneously the two absorption molecular transitions of CO_2 _and H_2_O. The laser frequency is swept over a range of ~20 GHz which includes the two absorption features of CO_2 _(line *R*(34) of the *ν*_1 _+ 2ν20
 MathType@MTEF@5@5@+=feaafiart1ev1aaatCvAUfKttLearuWrP9MDH5MBPbIqV92AaeXatLxBI9gBaebbnrfifHhDYfgasaacH8akY=wiFfYdH8Gipec8Eeeu0xXdbba9frFj0=OqFfea0dXdd9vqai=hGuQ8kuc9pgc9s8qqaq=dirpe0xb9q8qiLsFr0=vr0=vr0dc8meaabaqaciaacaGaaeqabaqabeGadaaakeaacqaIYaGmiiGacqWF9oGBdaqhaaWcbaGaeGOmaidabaGaeGimaadaaaaa@316A@ + *ν*_3 _combination band) and H_2_O (line 15_0,15 _→ 14_0,14 _of the *ν*_2 _+ *ν*_3 _band). The absorption spectra are acquired by means of a multifunction PCMCIA digital acquisition board (National Instruments NI DAQCard-6036E for PCMCIA), with a 16-bit analog-to-digital converter (ADC), at a maximal acquisition rate of 200 kS/s, interfaced to the LC. The temperature of the gas is also monitored in real time, with accuracy of 0.1°C, by acquiring the signal of a thermocouple via one of the ADC channels. A single absorption signal is acquired at the frequency scan of 2 Hz, so concentration values can be obtained every 0.5 s. Otherwise, it is possible to average several signal before processing the spectra and retrieve the concentration values.

The process is made in real time, even though all the spectra are stored for further off-line analysis. The acquisition program, written in Lab View^® ^language, controls the intruments settings and analyzes the acquired spectra. The fitting procedure described below is made by running a Matlab routine called by the main program.

### Signal analysis and preliminary tests

If *P*_0_(*ν*) is the power emitted by the laser as a function of the frequency *ν*, then the power *P*(*ν*) detected after passing through the absorbing cell is given, according to the Beer-Lambert law, by

P(ν)=P0(ν)exp⁡[ACO2g(ν−νCO2)+AH2Og(ν−νH2O)],
 MathType@MTEF@5@5@+=feaafiart1ev1aaatCvAUfKttLearuWrP9MDH5MBPbIqV92AaeXatLxBI9gBaebbnrfifHhDYfgasaacH8akY=wiFfYdH8Gipec8Eeeu0xXdbba9frFj0=OqFfea0dXdd9vqai=hGuQ8kuc9pgc9s8qqaq=dirpe0xb9q8qiLsFr0=vr0=vr0dc8meaabaqaciaacaGaaeqabaqabeGadaaakeaacqWGqbaucqGGOaakiiGacqWF9oGBcqGGPaqkcqGH9aqpcqWGqbaudaWgaaWcbaGaeGimaadabeaakiabcIcaOiab=17aUjabcMcaPiGbcwgaLjabcIha4jabcchaWjabcUfaBjabdgeabnaaBaaaleaacqqGdbWqcqqGpbWtdaWgaaadbaGaeGOmaidabeaaaSqabaGccqWGNbWzcqGGOaakcqWF9oGBcqGHsislcqWF9oGBdaWgaaWcbaGaee4qamKaee4ta80aaSbaaWqaaiabikdaYaqabaaaleqaaOGaeiykaKIaey4kaSIaemyqae0aaSbaaSqaaiabbIeainaaBaaameaacqaIYaGmaeqaaSGaee4ta8eabeaakiabdEgaNjabcIcaOiab=17aUjabgkHiTiab=17aUnaaBaaaleaacqqGibasdaWgaaadbaGaeGOmaidabeaaliabb+eapbqabaGccqGGPaqkcqGGDbqxcqGGSaalaaa@5F9E@

Here *g*(*ν *- *ν*_*X*_) is the unit-area normalized lineshape function, centred at corresponding line center frequency *ν*_*X *_for the transition *X*, which is assumed to be Lorentzian, as the gas is usually at atmospheric pressure:

g(ν−νX)=1πγX(ν−νX)2+γX2,
 MathType@MTEF@5@5@+=feaafiart1ev1aaatCvAUfKttLearuWrP9MDH5MBPbIqV92AaeXatLxBI9gBaebbnrfifHhDYfgasaacH8akY=wiFfYdH8Gipec8Eeeu0xXdbba9frFj0=OqFfea0dXdd9vqai=hGuQ8kuc9pgc9s8qqaq=dirpe0xb9q8qiLsFr0=vr0=vr0dc8meaabaqaciaacaGaaeqabaqabeGadaaakeaacqWGNbWzcqGGOaakiiGacqWF9oGBiiaacqGFsislcqWF9oGBdaWgaaWcbaacbaGae0hwaGfabeaakiabcMcaPiabg2da9maalaaabaGaeGymaedabaGae8hWdahaamaalaaabaGae83SdC2aaSbaaSqaaiabdIfaybqabaaakeaacqGGOaakcqWF9oGBcqGHsislcqWF9oGBdaWgaaWcbaGaemiwaGfabeaakiabcMcaPmaaCaaaleqabaGaeGOmaidaaOGaey4kaSIae83SdC2aa0baaSqaaiabdIfaybqaaiabikdaYaaaaaGccqGGSaalaaa@4ACA@

where *γ*_*X *_is the Half Width at Half Maximum (HWHM). *A*_*X *_is the integral area of the absorption feature which is the observable related to the gas concentration *N*_*X*_, according to

**Figure 1:**
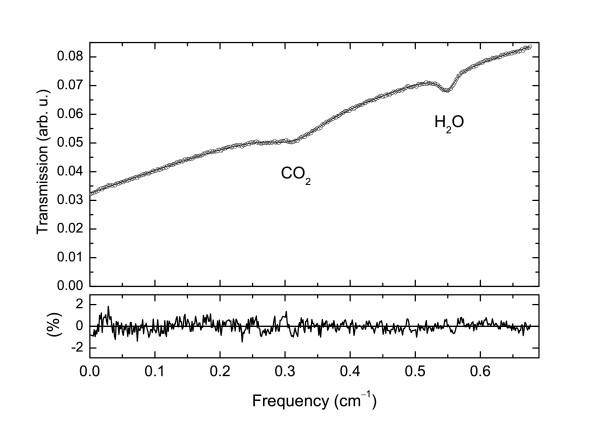
An example of an experimental absorption spectrum (○) and the best fit of Eq. (1) (solid line). In the plot below the residual between experimental data and the best fit is reported as percentage variation with respect to the signal. The standard deviation of the plotted residual is 0.5%.

*A*_*X *_= -*S*_*X*_(*T*) *LN*_*X*_,

where *S*_*X*_(*T*) is the line strength of the transition, which depends on the temperature of the sample through a known relation, and *L *is the absorption path length. The identification of the chosen spectral lines has been made according the spectroscopical database HITRAN [38], which also excluded the presence of other interfering gas species. The frequency scale has been previously determined by calibrating the time scale of the modulating ramp with a Fabry-Pérot cavity with known free spectral range [19]. Due to the long time stability of the laser source, this calibration has been shown to be stable over several months. Eq. (1) is fitted to the absorption spectrum according to the Levenberg-Marquardt method, and from the knowledge of the parameters resulting from the fit we can calculate the integral area *A*_*X *_of the absorption and finally retrieving, through Eq. (2), the concentration of the two species, knowing the two experimentally determined transition line strengths, SCO2
 MathType@MTEF@5@5@+=feaafiart1ev1aaatCvAUfKttLearuWrP9MDH5MBPbIqV92AaeXatLxBI9gBaebbnrfifHhDYfgasaacH8akY=wiFfYdH8Gipec8Eeeu0xXdbba9frFj0=OqFfea0dXdd9vqai=hGuQ8kuc9pgc9s8qqaq=dirpe0xb9q8qiLsFr0=vr0=vr0dc8meaabaqaciaacaGaaeqabaqabeGadaaakeaaieGacqWFtbWudaWgaaWcbaGaee4qamKaee4ta80aaSbaaWqaaiabikdaYaqabaaaleqaaaaa@316A@ = 4.77(3) × 10^-22 ^cm/mol at 301.4 K [19] and SH2O
 MathType@MTEF@5@5@+=feaafiart1ev1aaatCvAUfKttLearuWrP9MDH5MBPbIqV92AaeXatLxBI9gBaebbnrfifHhDYfgasaacH8akY=wiFfYdH8Gipec8Eeeu0xXdbba9frFj0=OqFfea0dXdd9vqai=hGuQ8kuc9pgc9s8qqaq=dirpe0xb9q8qiLsFr0=vr0=vr0dc8meaabaqaciaacaGaaeqabaqabeGadaaakeaacqWGtbWudaWgaaWcbaGaeeisaG0aaSbaaWqaaiabikdaYaqabaWccqqGpbWtaeqaaaaa@316D@ = 2.28(4) × 10^-24 ^cm/mol at 298 K [21], and the optical path length *L *= 20.3 m. The baseline has been assumed to be a second degree polynomial, as in laboratory condition it has been shown to well reproduce the signal when no absorption is present [20]. This baseline is very stable on a time basis of hours, as it is essentially determined by the increasing of the emitted power as the injection current of the laser increases during frequency scan. No effect of fringes was observed on baseline stability. During the field measurement environmental additional effect (i.e., sudden attenuation of signal from smoke or condensed vapour) could drastically change the baseline, but these cases were uncommon and were easily excluded by monitoring the *χ*^2 ^of the fit as a reliability parameter of the fit. Figure (1) shows a typical experimental absorption spectrum recorded during the present campaign, along with the best fit of Eq. (1). In the lower part it is also shown the residual in percentage between experimental signal and fitted curve, normalized with respect to the laser power. The error bar associated to each concentration value in the reported plots are determined by the propagation of the errors given by the fit for each parameters. For each fit the resulting *χ*^2 ^is monitored in order to verify the reliability of the results.

A preliminary laboratory survey has been performed on the spectrometer in order to verify its performances as well as the procedure to retrieve the gas concentrations.

Several tests were done using certified gas mixtures as reported in [19,21] with more details: the short term reproducibility over several minutes was found to be of 0.3% and 0.5% for CO_2 _and H_2_O, respectively. Long term measurements (24 hours) were also performed. The dynamic range of the spectrometer was fixed in the interval 30 – 10000 ppm, as mainly limited from the maximum acceptable relative uncertainty of 10% for the retrieved concentration (lower limit), and from the maximum frequency scan to be imposed on the laser (fixed to 1 cm^-1^) (upper limit) in order to keep the tails of the absorption within.

A comparisons between the performances of the laser spectrometer and a commercial infrared gas analyzer (IRGA) was also performed [19].

The robustness of the fitting procedure was verified with different values of the initial parameters values, as well as with Levenberg-Marquardt damping factors. Once the convergence in the fit procedure is achieved, the values of the retrieved concentration resulted not seriously biased neither by the initial values of the parameter, nor by the damping factors. Conversely the right choice of both these parameters turned out to be crucial in the number of iterations necessary to achieve convergence.

### Field measurements

**Figure 2:**
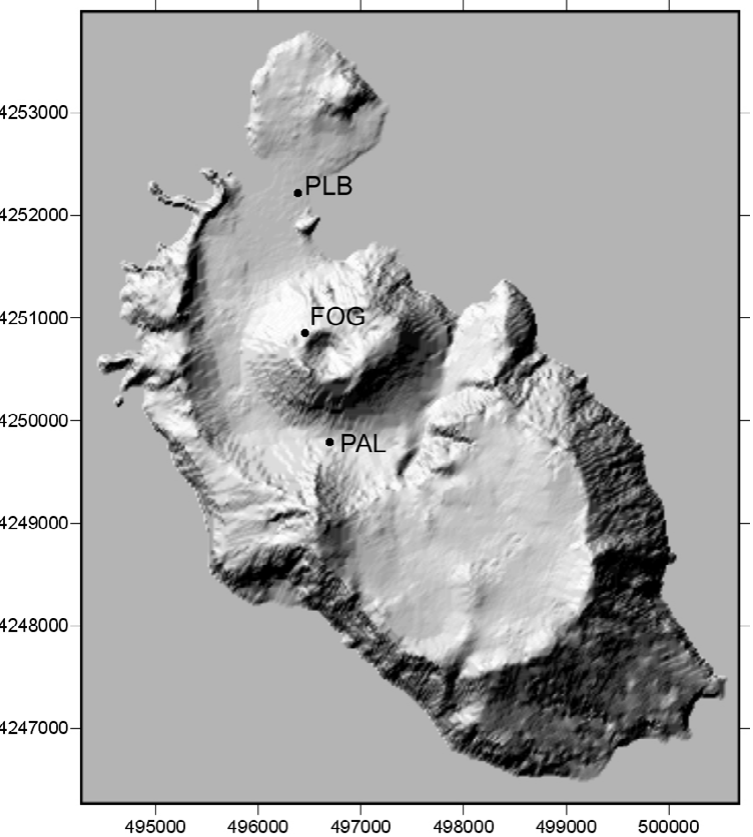
DTM (Digital Terrain Model) of the Vulcano island with the indication of the main measurement sites: PLB: Porto Levante Beach; FOG: Fossa Grande Crater; PAL: Valley of Palizzi.

The measurement campaign was held in August 25th–29th, 2004 at Vulcano island. During the campaign we analysed the emissions in different sites: PLB, PAL, and FOG, Fig. 2. These sites were choosen taking into account the previous studies [22-27,29,30,32-37,39-41] of the volcanic activity on Vulcano, identified as the extensive fumarole field in the northern part of La Fossa crater (100°C< T < 600°C), low-temperature fumaroles (T ≈ 100°C) in the Baia di Levante area, and widespreaded manifestations in the soil in the Vulcano Porto area and around the volcanic cone in the Palizzi valley. We performed measurements with two different configurations, with and without the breadboard cover, depending on the type of emissions we found and on the environmental conditions.

The first configuration was without the breadboard top cover, with the possibility of placing it at different heights from the ground. The second configuration was with the breadboard placed at the ground and with the top cover closed, in such a way to form an inverted accumulation chamber allowing the gas to flow from the soil to the sensing cell, through the aperture of the breadboard. The sealing of the base is ensured by the sligth burying of the breadboard in the soil. The latter was used when (1) there was no appreciable difference between the CO_2 _concentration and the average background value, if measured with an open configuration, and (2) there was low water vapour emission. Indeed, when concentration of water vapour in the accumulation chamber is so high that condensation of liquid water occurs on the colder surface of the mirrors and lenses, the final power on the detector shows strong attenuation, decreasing the signal to noise ratio and finally the sensitivity. The time to determine the flux is comparable with that of other accumulation chamber. However the sampled air is measured directly at the surface were the flux occurs, with no need of move the air with pumps to the measurement cell.

### Gas concentrations

Gas concentration measurements at Vulcano island have been performed at the three main sites (Fig. 2), namely PLB, FOG crater and PAL, and relevant data are resumed in Table 1. Different configurations were exploited.

**Table 1:** Mean values and standard deviations for CO_2 _and H_2_O concentrations and for their ratios, as measured for each site. The sites were labelled in the first column (see the text for details): measurement PLB1 to PLB3 were taken at different heigth from the ground level. All the remaining measurement were taken at ground level.

Site	T/°C	CO_2_/ppm			H_2_O/ppm			CO_2_/H_2_O		
		Mean	(error)	st.dev.	mean	(error)	st.dev.	mean	(error)	st.dev.
Porto Levante Beach (2/8/04)										

PLB1	26	769	(19)	240	39700	(400)	5600	0.0195	(5)	0.0058
PLB2	26	477	(13)	122	36200	(400)	3500	0.0132	(4)	0.0034
PLB3	26	450	(12)	115	34500	(400)	3600	0.0131	(3)	0.0032

Palizzi (26/8/04)										

PAL1	26	384	(3)	40	25540	(80)	1140	0.0150	(1)	0.0016
PAL2	26	468	(3)	44	25830	(75)	1050	0.0182	(1)	0.0018
PAL3	24	564	(12)	120	23130	(75)	740	0.0244	(5)	0.0053

Fossa Grande (27/8/04)										

FOG1	25	481	(24)	170	29000	(1000)	7500	0.0174	(12)	0.0090
FOG2	26	564	(15)	120	34300	(500)	3400	0.0165	(4)	0.0031
FOG3	29	541	(22)	200	35200	(2000)	19600	0.0195	(5)	0.0058
FOG4	26	505	(20)	173	30200	(900)	8000	0.0178	(3)	0.0074

**Figure 3:**
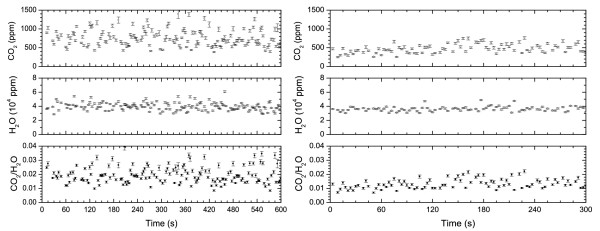
Two examples of concentration measurement at different heigth from the ground, 0 cm (left), 40 cm (right), made at the PLB (Aug 25th 2004) and corresponding to acquisition PLB1 and PLB3 of Table 1.

**Figure 4:**
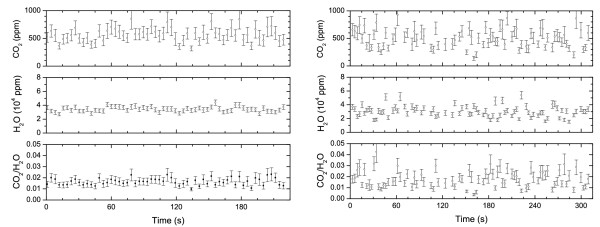
Two examples of measurements at FOG crater (Aug 27th 2004): left ≈ 5 meters away from the fumarole, right at the fumarole border corresponding to data FOG2 and FOG4 of Table 1, respectively.

**Figure 5:**
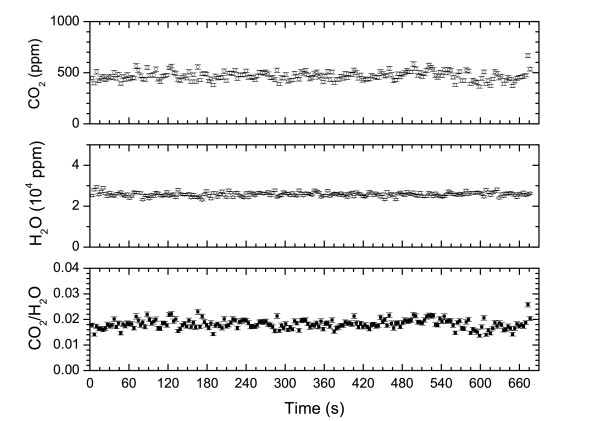
Concentration measurement at PAL(Aug 26th 2004). Corresponding to data PAL2 of Table 1.

Table 1 resumes the mean values and errors for CO_2_, H_2_O and their ratios at the different sampling points. Measurements at PLB (August 25th, 18:30 local time) have been performed setting the apparatus at various heights over the ground (PLB1 at 0 cm; PLB2 at 40 cm; PLB3 at 130 cm), in order to estimate the influence, on CO_2 _and H_2_O concentrations, due to the distance of the breadboard from the ground (Fig 3). Values of CO_2 _concentrations range from 1500 to 450 ppm, with average values measured decreasing from 779 ppm at ground to 450 ppm at 130 cm of height. The average value is rather stable from 44 to 130 cm, indicating that we are measuring, at these heights, almost the gas concentration in the atmosphere. H_2_O concentration is, on average, of about 4% at ground, slightly decreasing to 3.5% at 130 cm of height. We took also a measurement at 20 cm from the ground which however presented very noisy spectra, resulting in unreliable concentration values greater than those measured at ground level, which we cannot explain. Examples of measurements obtained at FOG (August 27th, 16:00) are shown in Fig. 4. At this site, four different points, at distances of few meters each other, have been sampled, in order to have an idea of the variability of results within small distances. Average values for CO_2 _concentrations range from 564 ppm to 481 ppm, whereas H_2_O ones are between 2.9% and 3.5% as shown in Table 1. At PAL site (August 26th, 17:00), three points within distances of some meters where selected for measurements, and examples are shown in Fig. 5 while results are reported in Table 1. Average values for CO_2 _concentrations lie in the range 384 to 564 ppm while for H_2_O, they are between 2.3% and 2.6%.

### Flux measurements with accumulation configuration

In the accumulation chamber configuration, the rate of change of the measured concentration can be modeled by the following differential equation,

dCc(t)dt=α[Cs−Cc(t)]
 MathType@MTEF@5@5@+=feaafiart1ev1aaatCvAUfKttLearuWrP9MDH5MBPbIqV92AaeXatLxBI9gBaebbnrfifHhDYfgasaacH8akY=wiFfYdH8Gipec8Eeeu0xXdbba9frFj0=OqFfea0dXdd9vqai=hGuQ8kuc9pgc9s8qqaq=dirpe0xb9q8qiLsFr0=vr0=vr0dc8meaabaqaciaacaGaaeqabaqabeGadaaakeaadaWcaaqaaiabdsgaKjabdoeadnaaBaaaleaacqWGJbWyaeqaaOGaeiikaGIaemiDaqNaeiykaKcabaGaemizaqMaemiDaqhaaiabg2da9GGaciab=f7aHjabcUfaBjabdoeadnaaBaaaleaacqWGZbWCaeqaaOGaeyOeI0Iaem4qam0aaSbaaSqaaiabdogaJbqabaGccqGGOaakcqWG0baDcqGGPaqkcqGGDbqxaaa@450A@

where *C*_*c *_and *C*_*s *_are the concentration of the CO_2 _in the chamber and in the soil, respectively, and *α *is the flux rate. Assuming a concentration *C*_*c*_(0) at the time *t *= 0 when the chamber is closed, the concentration in the chamber will change according to

*C*_*c*_(*t*) = *C*_*s *_- [*C*_*s *_- *C*_*c *_(0)]*e*^-*α*t^.

**Figure 6:**
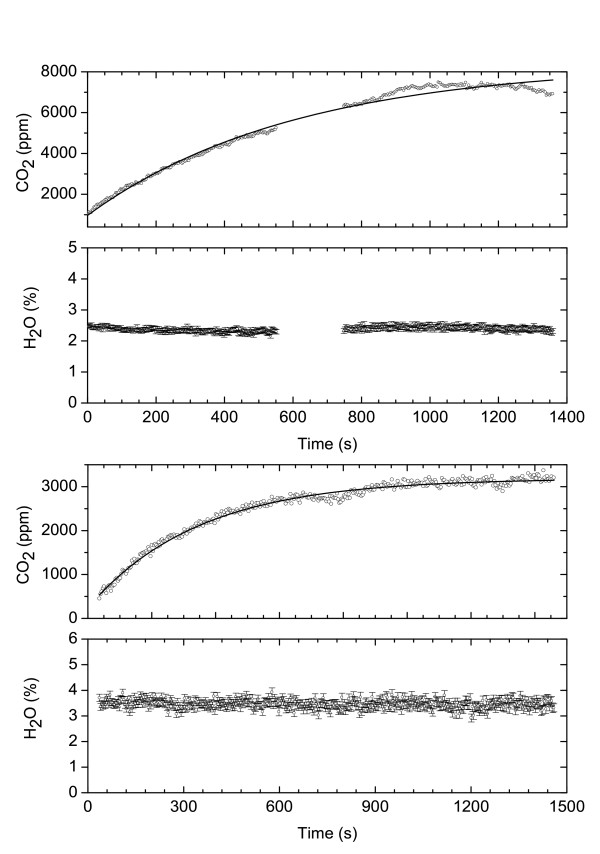
Flux meaurement at PAL – Aug 26th (above) and 28th (below) 2004. Solid lines represent the best fit of Eq. (3) to experimental data. Fluxes corresponding to data PAL Fl and PAL F2 of Table 2.

By fitting the last expression to the experimental data we can retrieve the flux rate *α *and the concentration *C*_*s*_. Examples of fitting procedure are shown in Fig. 6. The retrieved fit parameters are listed in Table 2. Knowing the volume of the chamber and the open area at base of the chamber, we can estimate the mass flux at the soil, as reported in the last column of Table 2.

**Table 2:** Accumulation measurements. Concentration rate *α *and saturation values *C*_*s *_retrieved by fitting Eq. (3) to the experimental data. In the last column we list the respective flux of CO_2 _(at Standard Temperature and Pressure). The sites were labelled in the first column (see the text for details).

Site	T/°C	*α*/10^-3^s^-1^	*C*_*s*_/ppm	flux/g m^-2^s^-1^
Palizzi (26/8/04)				

PAL F1	24	1.65 (4)	8390 (60)	1.73

Palizzi (28/8/04)				

PAL F2	36	3.06 (6)	3140 (10)	3.20
PAL F3	40	0.90 (6)	6750 (270)	0.94
PAL F4	34	1.82 (10)	12950 (440)	1.90

Porto Levante Beach (28/8/04)				

PLB F1	33	1.72 (6)	28330 (510)	1.80

Measurements of CO_2 _flux at three sites of PAL (August 28th, 18:00) resulted in rates of 0.0031, 0.00090, 0.0018 s^-1 ^(Table 2). At the first site, in a measurement took two days before(August 26th, 17:00), we get a rate of 0.0017 s^-1 ^(Fig. 6). The concentration of water vapour remained constant during each flux measurement and its value is the same in the three different measurements of day August 28th, while for the two measurements made in the same place but in two different days (August 26th and 28th) we observed a change in water vapour concentration. This suggests that the presence of water is merely due to umidity of air. A measurements at Porto Levante Beach (August 29, 11:00)gave a flux rate of 0.0017 s^-1^. During this measurement we observed an increase of water vapour concentration, however we believe it is likely due to the evaporation of water soil content due to sun at the measuring site after raining. Flux measurements at FOG, at distance of few meters from the main fumarole, showed not detectable flux (Table 2). Similar accumulation measurements in the fumarole failed for the condensation of water vapour on the mirrors which reduced the signal to zero.

## Conclusion

We have carried out a measurement campaign in Vulcano island with a portable spectrometer based on a semiconductor laser, which can monitor in-situ concentration and flux of CO_2 _and H_2_O. The spectrometer has a response time of the order of one second which allows the monitoring of gas dynamics on a very fast time scale. The measurements obtained at Vulcano, together with several tests, performed continuosly for several days, indicate the system has no problem to be used for long-time unattended measurements as well. The only occasional problems for a long-term remote installation could be the condensation of water vapour on the mirrors; such problem could be be reduced by heating the mirrors' surface. The average values of the CO_2_/H_2_O at ground level of the different measurement sites (0.0195–0.0150) is consistent with an average value measured for example at the FO fumarole using different comparative methods (FTIR, Filter pack and direct sampling) of about 0.063 [22]. Accumulation measurements have shown the presence of non-negligible CO_2 _flux from the soil, at several places, which can be missed by simply monitoring the concentration in air. The measurements of gas concentration and CO_2 _fluxes carried out during the present field work at Vulcano have demonstrated that laser diode spectrometers represent a suitable choice for monitoring areas with anomalous high gas concentration, in different environmental conditions. Our apparatus has shown to be suitable for continuous, in-situ monitoring of both gas concentrations and fluxes, with laboratory precision. Also, it can be considered a reliable instrument useful to the local community in areas subjected to dangerous episodes of CO_2 _exhalation from soils (i.e., Ciampino and Marino towns in the Albani Hills, Central Italy [42]; Mefite d'Ansanto close to Avellino city, Southern Italy; Mammuth area Long Valley, California USA [43]; Lake Nyos and Monoun, Cameroon [44]).
